# Causal associations between type 2 diabetes mellitus, glycemic traits, dietary habits and the risk of pressure ulcers: univariable, bidirectional and multivariable Mendelian randomization

**DOI:** 10.3389/fnut.2024.1375179

**Published:** 2024-10-02

**Authors:** Pei Luo, Can Huang

**Affiliations:** ^1^Department of Thoracic Surgery, Tangdu Hospital, Air Force Medical University, Xi'an, Shaanxi, China; ^2^Department of Cardiovascular Surgery, Peking University Shenzhen Hospital, Shenzhen, China

**Keywords:** pressure ulcers, dietary habits, glycemic traits, Mendelian randomization, type 2 diabetes, multivariate

## Abstract

**Objective:**

Previous research has established a connection between Type 2 Diabetes Mellitus (T2DM), glycemic traits, dietary habits, and the risk of Pressure Ulcers (PUs). The aim of our study is to disentangle any potential causal relationship between T2DM, glycemic traits, and dietary factors, and the risk of PUs.

**Methods:**

The exposure and outcome datasets were sourced from the IEU Open GWAS project, the Meta-Analyses of Glucose and Insulin-related traits Consortium (MAGIC), and the FinnGen biobank, respectively. The primary MR analysis method employed was the inverse variance-weighted method. Furthermore, we employed multivariable MR (MVMR) adjusting for BMI. Then, we investigated the possibility of a reverse association between glycemic traits and PUs through bidirectional MR. Finally, Heterogeneity and pleiotropic analysis were conducted to ensure the accuracy and robustness of the results.

**Results:**

The findings revealed that T2DM (OR = 1.282, 95% CI: 1.138–1.445, *p* < 0.001) and Fasting Glucose (FG; OR = 2.111, 95% CI: 1.080–4.129, *p* = 0.029) were associated with an increased risk of PUs, while salad/raw vegetable intake (OR: 0.014; 95% CI: 0.001–0.278; *p* = 0.005) was identified as a protective element. However, no other dietary elements demonstrated a statistically significant causality with PUs. In addition, in the reverse direction, there were positive correlation between genetic susceptibility to PUs and an increase in FG (OR: 1.007, 95% CI: 1.000–1.013, *p* = 0.048) and Fasting Insulin (FI; OR: 1.012, 95% CI: 1.003–1.022, *p* = 0.011). MVMR results indicated that the causal effect of T2DM on PUs was independent of BMI (OR: 1.260, 95% CI: 1.112–1.427, *p* < 0.001). These results remained robust when considering weak instrument bias, pleiotropy, and heterogeneity.

**Conclusion:**

This study establishes a causal link between genetically predicted T2DM, FG and an increased risk of PUs. Conversely, Salad/raw vegetable intake is significantly inversely associated with PUs. Simultaneously, we identified two downstream effector factor (FG and FI) that were associated with PUs. These findings may have clinical implications for both prevention and treatment.

## 1 Introduction

Pressure ulcers (PUs), also known as pressure sores, bed sores or decubitus ulcer, are localized injures to the skin and underlying tissue. They typically occur over bony prominences, and are classified into stages (Stage 1 to Stage 4) based on their severity (1, 2). These injuries develop when prolonged pressure is applied to the skin, often in combination with friction and shear forces. PUs are a significant concern in healthcare, particularly for individuals with limited mobility, such as those who are bedridden or use wheelchairs (3). PUs have substantial healthcare and economic implications and are present in all healthcare settings (4). Although most PUs are reasonably preventable (5), approximately 1 to 3 million individuals in the United States develop PUs each year (6), and 60,000 people die each year due to complications from PUs (7). The management costs of PU are a major problem, with a recent study estimated the annual cost in the United States to be in excess of $26.8 billion (8), and in the United Kingdom, it is estimated at £1.4–£2.1 billion *per annum* (9). Moreover, PUs can significantly diminish overall quality of life due to pain, the need for management procedures, extended hospital stays, and psychosocial burdens (2, 10, 11). Therefore, identifying the risk and protective factors for PUs is crucial for its prevention and management.

In developing countries and low-income households in the United States, individuals face not only a financial burden but also a higher risk of negative effects because proper nutrition or caregiver support may be deficient (12–14). Insufficient dietary habits or malnutrition may play an important role in this increase of PUs. The pathogenesis of PUs is multifactorial and may be related to genetic, environmental, and lifestyle factors. A number of studies have revealed that inadequate nutritional intake is associated with a higher risk of developing PUs (15–17). In particular, dietary patterns characterized by elevated fiber content, notably derived from whole grains, fruits, and vegetables, have demonstrated an association with enhanced glycemic control. The presence of fiber in these diets serves to decelerate the absorption of glucose, thereby mitigating spikes in blood sugar levels. Conversely, the consumption of a diet rich in added sugars and processed foods has been implicated in the elevation of blood glucose levels, potentially fostering insulin resistance. Individuals exhibiting heightened fasting blood sugar levels or impaired glucose tolerance face an augmented susceptibility to the onset of type 2 diabetes (T2DM) (18, 19). T2DM, a persistent disorder marked by compromised regulation of blood glucose, holds the potential to manifest as enduring microvascular and macrovascular complications and accumulating evidence showed that patients with type 2 diabetes mellitus (T2DM) are 1.5 to 2 times more likelihood to develop surgery-related PUs than patients with normal glucose tolerance (20, 21). The previous studies on potential risk factors for PUs are based on observational research, which may be susceptible to issues related to potential residual confounding and reverse causation (22). We now aim to assess the causal association between dietary habits, T2DM, and the likelihood of developing PUs using Mendelian randomization (MR).

Under certain assumptions, MR utilizes genetic variants as instrumental variables (IVs), offer the advantage of controlling for nonheritable confounders and reverse causation (23). To date, there has been no genetic study on the relationship between T2DM, glycemic traits, dietary habits, and PUs. Herein, we performed a two-sample MR study to explore the causal relationship between dietary habits, T2DM and PU in this study.

## 2 Methods

**Figure 1:**
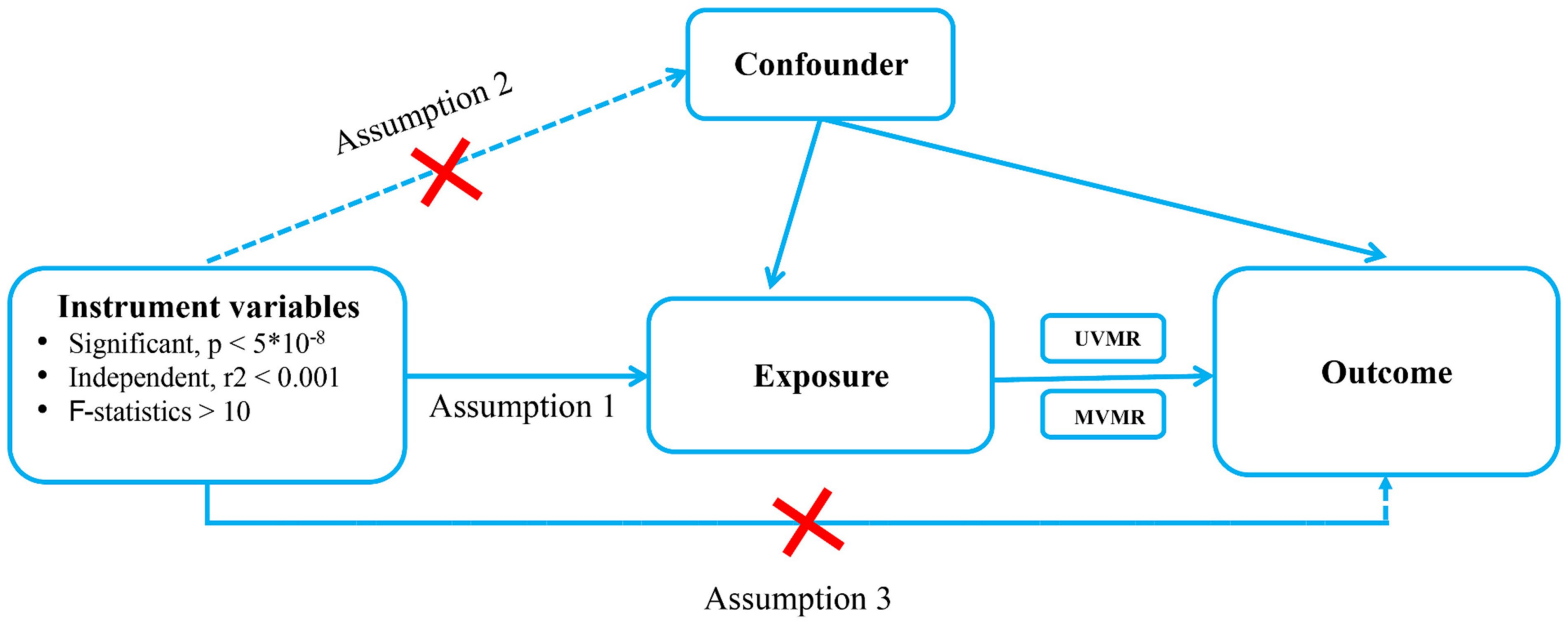
Schematic showing how Mendelian randomization was used to evaluate a causal association between T2DM, glycemic traits, Dietary habits and pressure ulcers in this study. T2DM, Type 2 diabetes Mellitus; UVMR, Univriable mendelian randomization; MVMR, multivariable mendelian randomization.

The following basic assumptions constitute the premise of MR analysis. (1) IVs must be directly associated with the exposures; (2) IVs cannot be directly correlated to the outcome only via exposure but not through other pathways; (3) IVs were independent of any potential confounding factors (Figure 1). The GWAS summary-level data used in this study were issued by the IEU open GWAS project,^1^ MAGIC^2^ and FinnGen biobank (FREEZE 9).^3^ This study was exempt from the approval of the Ethical Review Authority because the data used in this study was public, anonymized, and de-identified.

### 2.1 Data sources

We obtained genome-wide associations for 17 dietary habits, glycemic traits and a T2DM, derived from principal component (PC) analysis, by using UKBB GWAS summary statistics from Benjamin Neale’s lab,^4^ MAGIC and Xue et al. (24, 25), respectively. Glycemic traits and diet-associated exposure factors used in this study included fasting glucose (FG), 2 h-glucose post-challenge (2hGlu), glycated hemoglobin (HbA1c), and fasting insulin data (FI), Bread intake, Cheese intake, Drink (Alcoholic drinks per week, Alcohol intake frequency, Water intake, Coffee intake, and Tea intake), Fruit (Dried fruit intake and Fresh fruit intake), Cereal intake, Meat and Poultry (Beef intake, Pork intake, Processed meat intake, and Poultry intake), Seafood (Non-oily fish intake and Oily fish intake), and Vegetable (Salad / raw vegetable intake and Cooked vegetable intake). The GWAS summary-level data of PUs was extracted from FinnGen biobank. We did not use proxy single nucleotide polymorphisms (SNPs) when finding SNPs from the outcome, mainly because the FinnGen biobank contained enough SNPs (16,380,176 SNPs in the dataset of PUs). More information about the exposure and outcome datasets is presented in Table 1.

**Table 1:** Summary of the genome-wide association studies (GWAS) included in this two-sample MR study.

ID or PMID	Types	Exposures/outcomes	Sample size	Case control	SNPs (N)	Consortium (Author)	Population	Year
ebi-a-GCST006867	T2DM	Type 2 Diabetes	61,714	1,178	9,851,867	MRC-IEU	European	2018
MAGIC1000G_2hGlu_EUR	Giycemic traits	2 h glucose	452,236	NA	30,098,704	MAGIC	European	2021
MAGIC1000G_FG_EUR	Giycemic traits	fasting glucose	NA	NA	34,064,006	MAGIC	European	2021
MAGIC1000G_FI_EUR	Giycemic traits	fasting insulin	NA	NA	32,635,792	MAGIC	European	2021
MAGIC1000G_HbA1c_EUR	Giycemic traits	hemoglobin A1c	NA	NA	33,811,879	MAGIC	European	2021
ukb-b-11348	Bread	Bread intake	452,236	NA	9,851,867	MRC-IEU	European	2018
ukb-b-1489	Dairy Products	Cheese intake	451,486	NA	9,851,867	MRC-IEU	European	2018
ieu-b-73	Drink	Alcoholic drinks per week	335,394	NA	11,887,865	MRC-IEU	European	2019
ukb-b-5779	Drink	Alcohol intake frequency	462,346	NA	9,851,867	MRC-IEU	European	2018
ukb-b-14898	Drink	Water intake	427,588	NA	9,851,867	MRC-IEU	European	2018
ukb-b-5237	Drink	Coffee intake	428,860	NA	9,851,867	MRC-IEU	European	2018
ukb-b-6066	Drink	Tea intake	447,485	NA	9,851,867	MRC-IEU	European	2018
ukb-b-3881	Fruit	Fresh fruit intake	446,462	NA	9,851,867	MRC-IEU	European	2018
ukb-b-15926	Grains, Nuts, and Seeds	Cereal intake	441,640	NA	9,851,867	MRC-IEU	European	2018
ukb-b-2862	Meat and Poultry	Beef intake	461,053	NA	9,851,867	MRC-IEU	European	2018
ukb-b-5640	Meat and Poultry	Pork intake	460,162	NA	9,851,867	MRC-IEU	European	2018
ukb-b-6324	Meat and Poultry	Processed meat intake	461,981	NA	9,851,867	MRC-IEU	European	2018
ukb-b-8006	Meat and Poultry	Poultry intake	461,900	NA	9,851,867	MRC-IEU	European	2018
ukb-b-17627	Seafood	Non-oily fish intake	460,880	NA	9,851,867	MRC-IEU	European	2018
ukb-b-2209	Seafood	Oily fish intake	460,443	NA	9,851,867	MRC-IEU	European	2018
ukb-b-1996	Vegetable	Salad / raw vegetable intake	435,435	NA	9,851,867	MRC-IEU	European	2018
ukb-b-8089	Vegetable	Cooked vegetable intake	448,651	NA	9,851,867	MRC-IEU	European	2018
Ieu-b-40	Body mass index	Body mass index	681,275	NA	2,336,260	MRC-IEU	European	2018
finngen_R9_ L12_DECUBITANSULCERANDPRESSURE	PUs	Pressure ulcers	354,567	353,088	20,169,702	FinnGen	European	2022

SNP, Single nucleotide polymorphisms.

### 2.2 The selection of IVs

In MR analysis, IVs were utilized as mediators between exposure factors and outcomes to explore the causal relationship between exposure and outcomes. IVs are generally genetic variations, among which SNPs are the most commonly used. SNPs associated with dietary factors were extracted from the IEU open GWAS project. we screened the SNPs intensely related with exposures at the genome-wide significance level (*p* < 5 × 10^−8^), clumping window >10,000 kb, and the linkage disequilibrium level (r2 < 0.001). The F statistic greater than 10 was generally considered to meet the requirements of strong association. we searched the remaining SNPs for their associations with other phenotypes (such as body mass index and overweight (26, 27)) in PhenoScanner^5^ (28), a database of human genotype–phenotype associations and excluded those associated with potential confounding traits. However, in the reverse MR analysis, since very few SNPs were identified for part of PUs when they were as the exposure, a higher cutoff (*p* < 5e–06) was condisered as the genome-wide significant threshold.

### 2.3 Statistical analysis

We employed the random-effects inverse variance-weighted (IVW) method as the primary approach for calculating the causal effect. The IVW model is recognized for its strong capability in detecting causation in the two-sample MR analysis (18). To enhance the robustness of our findings, we compared the results obtained from the three different IVW methods, random-effects, fixed-effects, and multiplicative random-effects model, with those from the Weighted median, Simple mode, Weighted mode, and MR-Egger methods. The Weighted median method allows no more than 50% of invalid IVs, Simple Mode selects IVs with the most frequent occurrence in MR analysis, Weighted Mode, akin to Simple Mode, considers the weight of IVs, with higher frequency IVs receiving greater weights to enhance their contribution to causal estimation, while the MR-Egger method allows all IVs to be potentially invalidated (29). Thus, when all models yield consistent results, the evidence becomes more compelling.

We assessed heterogeneity in the IVW model using Cochran’s Q test, where a *p*-value of <0.05 indicates the presence of heterogeneity. It is important to note that the presence of heterogeneity does not necessarily invalidate the IVW model. Additionally, we utilized the MR-Egger method, which accommodates non-zero intercepts, to detect directional pleiotropy. To ensure the robustness of our results, we conducted a leave-one-out analysis to examine whether the removal of a SNP significantly influenced the outcomes. The MR-PRESSO method was employed to identify and address outliers. Upon detection of outliers, they were promptly removed, and the MR analysis was performed again.

Considering that BMI confound T2DM and glycemic traits in the pathogenesis of PUs, we used a multivariate MR approach to adjust for BMI, aiming to obtain an independent causal efect of T2DM and glycemic traits on the pathogenesis of PUs (30). The conditional F statistic for the BMI phenotype was calculated to evaluate the joint strength of instruments in the multivariable framework. All analyses were conducted using the TwoSampleMR package (version 0.5.7) (31), ‘MR-PRESSO’ package (version 1.0), ‘MendelianRandomization’ package (version 0.8.0) and ‘MVMR’ package (version 0.4) based on R software (version 4.2.2).

## 3 Results

### 3.1 Genetic instruments for T2DM and glycemic traits

SNPs with low allele frequencies <0.01 or no meaningful genome-wide association evidence (*p* < 5 × 10^−8^) were excluded. We identified 64, 10, 40, and 9 SNPs as LD-independent IVs (after the clumping process) in T2DM, 2hGlu, FG, FI, and HbA1c. In our study, F statistics were all significantly >10, suggesting that our results were highly trustworthy and largely unaffected by weak IVs (Supplementary Table S1).

### 3.2 Genetic instruments for 17 dietary habits

Supplementary Table S1 provides comprehensive information about each of the participating GWAS study. The analyses encompassed a total of 17 different dietary habits as exposures. The number of SNPs considered for each dietary habit varied between 3 and 60. We evaluated the influence of various dietary exposures on the outcome, and, in all cases, the F statistics of the identified SNPs exceeded the empirical threshold of 10, with the sum of *F* values ranging from 74.427 to 5216.822. This finding indicates that the results obtained are less susceptible to biases stemming from weak IVs.

### 3.3 Causal effects of T2DM, glycemic traits, and dietary habits on PU

**Figure 2:**
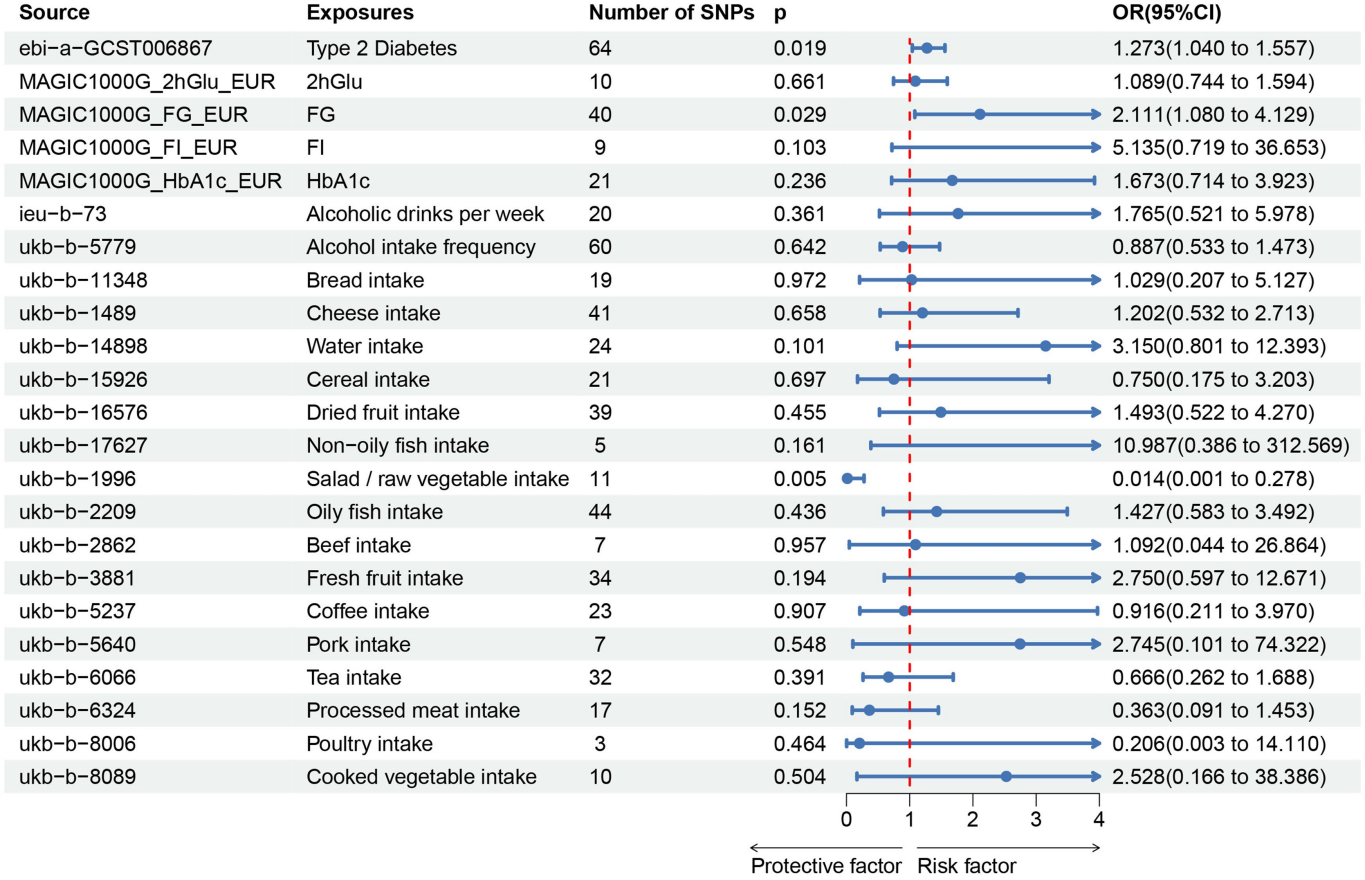
Associations of genetically predicted T2DM, glycemic traits, dietary habits with the risk of pressure ulcers. T2DM, Type 2 Diabetes Mellitus; FG, fasting Glucose; FI, Fasting Insulin; 2hGlu, 2 h-glucose post-challenge; HbA1c, glycated hemoglobin; and fasting insulin data (FI).

**Figure 3:**
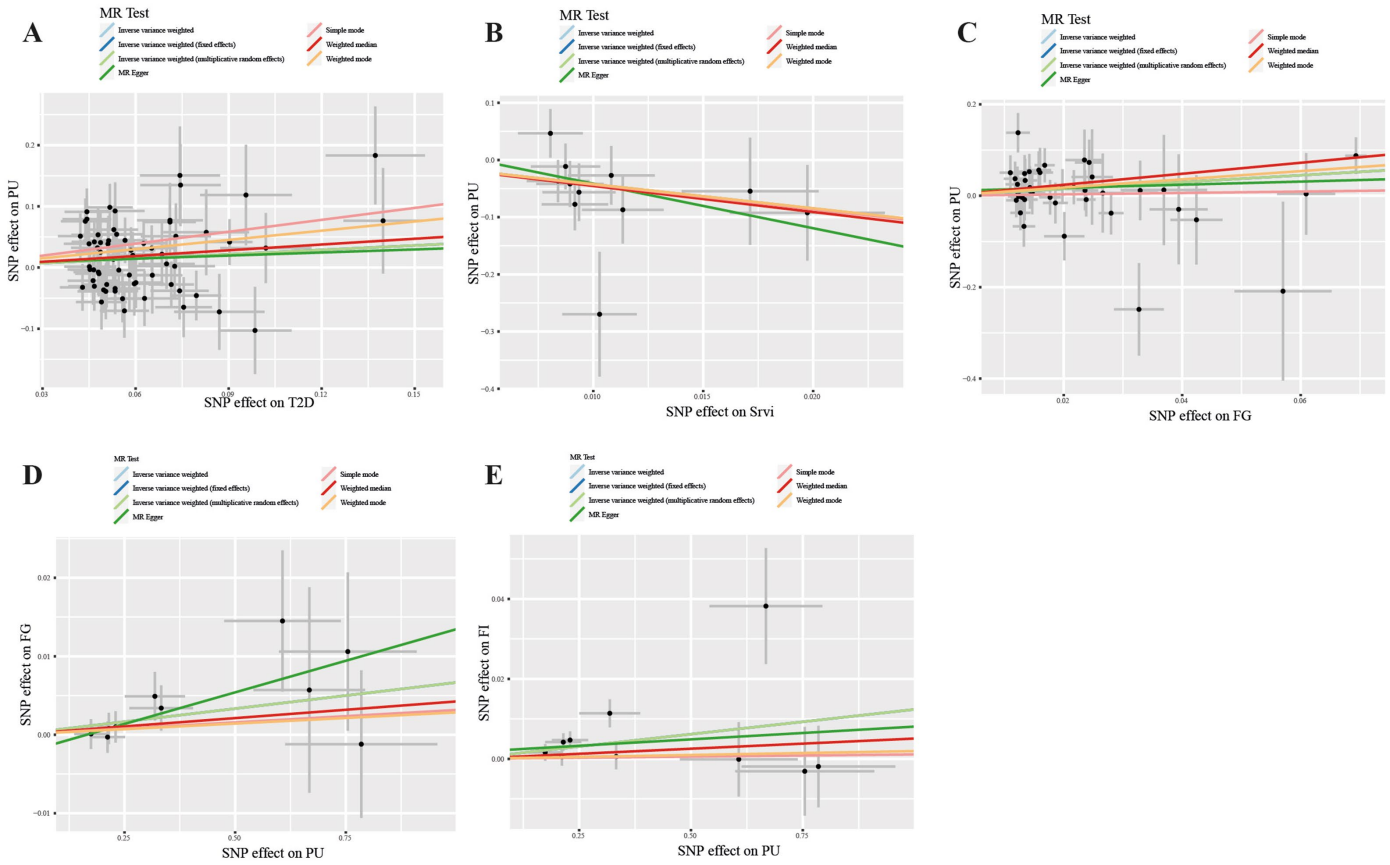
Scatter plot in the Mendelian randomization analysis of T2DM, salad/raw vegetable intake, glycemic traits and Pressure ulcers. Scatter plot of T2DM **(A)**, Srvi **(B)**, and FG **(C)** on PUs and PUs on FG **(D)** and FI **(E)**. T2DM, Type 2 Diabetes Mellitus; Srvi, salad/raw vegetable intake; PUs, Pressure ulcers; FG, fasting Glucose; FI, Fasting Insulin.

**Figure 4:**
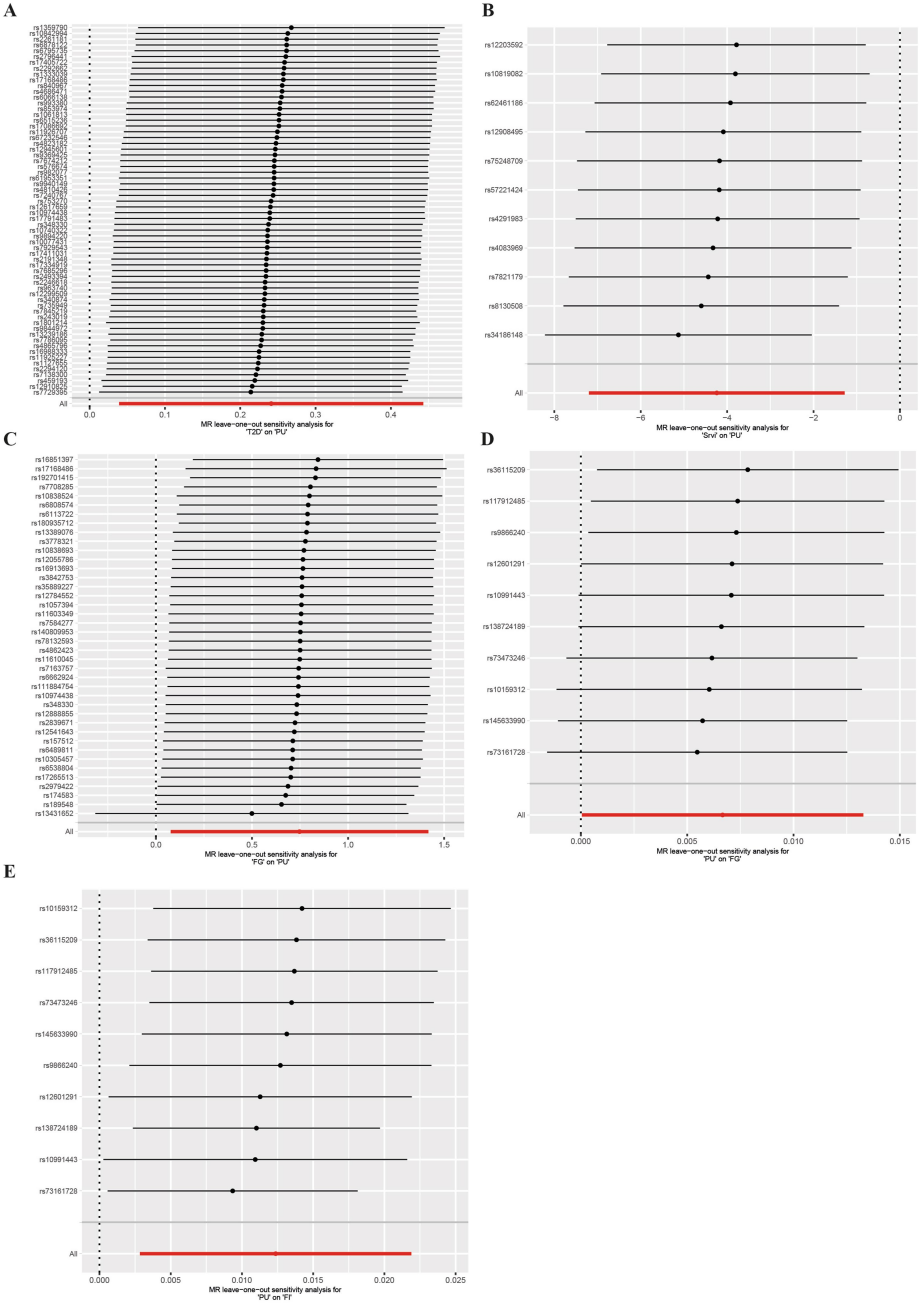
Leave-one-out sensitively analysis Scatter plot in the Mendelian randomization analysis of T2DM, salad/raw vegetable intake, glycemic traits and Pressure ulcers. “Leave-one-out” plot of T2DM **(A)**, Srvi **(B)**, and FG **(C)** on PUs and PUs on FG **(D)** and FI **(E)**. T2DM, Type 2 Diabetes Mellitus; Srvi, salad/raw vegetable intake; PUs, Pressure ulcers; FG, fasting Glucose; FI, Fasting Insulin.

In our study, a total of 3 causal associations were identified (*p* < 0.05 by IVW method). We found that T2DM (OR = 1.273, 95% CI: 1.040–1.557, *p* = 0.019) and FG (OR = 2.111, 95% CI: 1.080–4.129, *p* = 0.029) was related to an increased risk of PU, these discoveries were further verified by the consequences of the weighted median model (OR: 1.369; 95% CI: 1.045–1.793; *p* = 0.023) and (OR: 3.334; 95% CI: 1.235–9.000; *p* = 0.017), respectively. In contrast, salad/raw vegetable intake (OR: 0.014; 95% CI: 0.001–0.278; *p* = 0.005) was discovered as protective factors, and there was a statistically significant result in weighted median model (OR: 0.011; 95% CI: 0.000–0.498; *p* = 0.021). However, our study also indicated that Alcoholic drinks per week (OR = 1.765, 95% CI: 0.521–5.978, *p* = 0.361), Alcohol intake frequency (OR = 0.887, 95% CI: 0.533–1.473, *p* = 0.642), Bread intake (OR = 1.029, 95% CI: 0.207–5.127, *p* = 0.972), Cheese intake (OR = 1.202, 95% CI: 0.532–2.713, *p* = 0.658), Water intake (OR = 3.150, 95% CI: 0.801–12.393, *p* = 0.101), Cereal intake (OR = 0.750, 95% CI: 0.175–3.203, *p* = 0.697), Dried fruit intake (OR = 1.493, 95% CI:0.522–4.270, *p* = 0.455), Non-oily fish intake (OR = 10.987, 95% CI: 0.386–312.569, *p* = 0.161), Oily fish intake (OR = 1.427, 95% CI: 0.583–3.492, *p* = 0.436), Beef intake (OR = 1.092, 95% CI: 0.044–26.864, *p* = 0.957), Fresh fruit intake (OR = 2.750, 95% CI: 0.597–12.671, *p* = 0.194), Coffee intake (OR = 0.916, 95% CI:0.211–3.970, *p* = 0.907), Pork intake (OR = 2.745, 95% CI: 0.101–74.322, *p* = 0.548), Tea intake (OR = 0.666, 95% CI: 0.262–1.688, *p* = 0.391), Processed meat intake (OR = 0.363, 95% CI: 0.091–1.453, *p* = 0.152), Poultry intake (OR = 0.206, 95% CI: 0.003–14.110, *p* = 0.464), and Cooked vegetable intake (OR = 2.528, 95% CI: 0.166–38.386, *p* = 0.504) were not associated with PU in the IVW method (Figure 2; Supplementary Table S2). Albeit with there were no evidence for significant outliers, heterogeneity effect and potential pleiotropy (*p* > 0.05; Supplementary Tables S3, S4), we still used the fixed effect and multiplicative random effects IVW method as the major complementary approaches. The results of above two IVW methods remained the consistence with the random effect IVW. Moreover, scatter plots (Figures 3A–C) and funnel plots (Supplementary Figures S1A–C) confirmed the credibility of the results of our MR study. Based on the leave-one-out sensitively analysis (Figures 4A–C) and forest plot (Supplementary Figures S2A–C), it is suggested that the causal effect of T2DM and dietary habits on PUs was not driven by any single SNP.

### 3.4 Causal effects of PU on T2DM and glycemic traits

**Figure 5:**
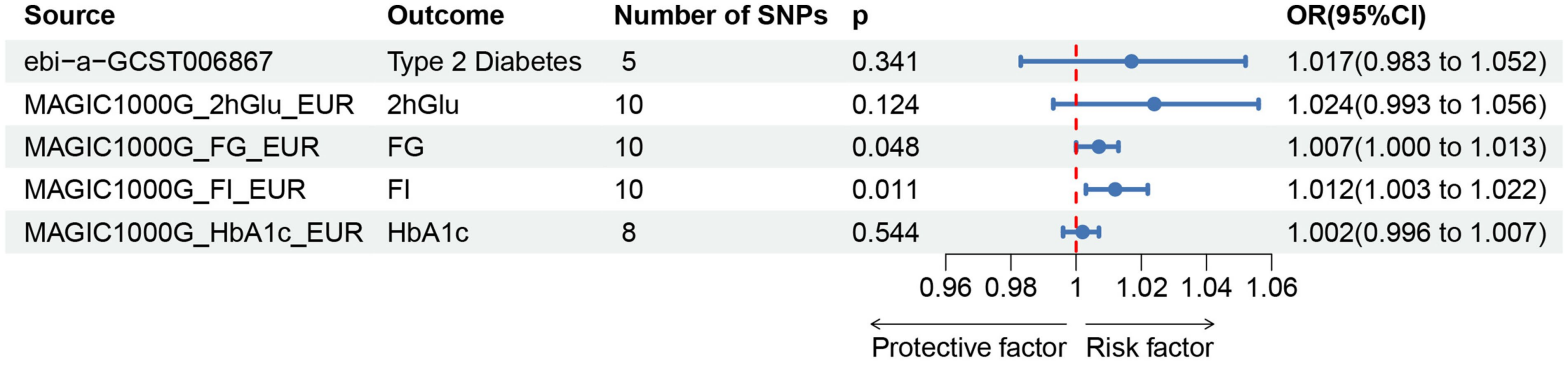
Associations of genetically predicted pressure ulcers on T2DM and glycemic traits.T2DM, Type 2 Diabetes Mellitus; FG, fasting Glucose; FI, Fasting Insulin; 2hGlu, 2 h-glucose post-challenge; HbA1c, glycated hemoglobin; and fasting insulin data (FI).

For the reverse MR analysis, a total of 10 SNPs implicated with PU were selected at a less stringent cut-off (*p* < 5 × 10^−6^), and all of which had F-statistics more than 10, demonstrating the positive association between genetically predicted PU patients and the levels of FG (OR: 1.007; 95% CI: 1.000–1.013; *p* = 0.048) and FI (OR: 1.012; 95% CI: 1.003–1.022; *p* = 0.011), the findings of other MR approaches were similar to the IVW method. In addition, no evidence showed the causal influence of PUs patients on T2DM (OR: 1.017; 95% CI: 0.983–1.052; *p* = 0.341), 2hGlu (OR: 1.024; 95% CI: 0.993–1.056; *p* = 0.124), and HbA1c (OR: 1.002; 95% CI: 0.996–1.007; *p* = 0.544; Figure 5; Supplementary Table S5). MR-PRESSO determined no horizontal pleiotropy, and no heterogeneity was observed in estimating the effect of PU patients on T2DM and Glycemic (Supplementary Tables S6, S7). Furthermore, our MR study results were supported by scatter plots (Figures 3D,E) and funnel plots (Supplementary Figures S1D,E), indicating credibility. The leave-one-out sensitivity analysis (Figures 4D,E) and forest plot (Supplementary Figures S2D,E) suggest that the causal effect of T2DM and dietary habits on pressure ulcers is not influenced by any single SNP.

### 3.5 Multivariate MR

To confirm whether the correlation of T2DM or FG on PUs is independent of BMI, the multivariate MR analysis was conducted. Specifically, we identified SNPs associated with T2DM or FG and BMI separately (*p* < 5 × 10^−8^) and combined all genetic variants. Following the exclusion of duplicate IVs, we observed a significant causal association between T2DM and BMI and PUs (*p* < 0.001; *p* = 0.026. respectively) but no causal association for FG (*p* = 0.432; Table 2).

**Table 2:** Multivariable MR results for T2DM or FG on PUs after adjusting for BMI.

Exposure	Outcome	SNP	Conditional *F*-value	*p*-value	OR (95% CI)
T2DM + BMI					
T2DM	PUs	56	15.950	<0.001	1.260 (1.112–1.427)
BMI	PUs	374	37.544	0.026	1.378 (1.039–1.829)
FG + BMI					
FG	PUs	461	5.651	0.432	1.427 (0.589–3.468)
BMI	PUs	375	71.987	0.257	1.151 (0.903–1.467)

SNP, Single nucleotide polymorphisms. OR, Odd ratios; CI, Confidence interval; T2DM, Type 2 Diabetes Mellitus; BMI, Body mass index; FG, fasting Glucose; PUs, Pressure ulcers.

## 4 Discussion

Based on summary level data from large GWAS, we implemented a two-sample univariable MR study to investigate the causal association between dietary habits and PUs. Employing a bidirectional analytical approach for T2DM, Glycemic traits, and the risk of PUs. We further applied Multivariable MR to eliminate the potential confounding factors, specifically BMI, revealing that T2DM is the independent effect factor on PUs, allowing us to differentiate between upstream and downstream factors in the disease pathway. Our results indicated that genetically predicted T2DM and FG are positively associated with the risk of PUs, while Salad / raw vegetable intake shows a negative association with PU risk. Additionally, we observed that PUs is associated with increased levels of FG and FI. These findings were generally robust in sensitivity analysis, ensuring the reliability of our MR analyses and mitigating potential pleiotropic effects. According to our knowledge, this represents the first MR study evaluating potential causative links between T2DM, glycemic traits, dietary habits, and PUs.These findings provide valuable insights for the prevention and treatment of PUs.

Our findings reveal that T2DM and the elevated levels of FG increase the risk of PUs, this conclusion aligns with the results of numerous studies. A meta-analysis of 15 observational studies, encompassing 19,724 intraoperative PUs in patients conducted in Asia, the Americas, Europe, and Australia from 1989 to 2019, indicated that the incidence of PUs in the T2DM population increased by 50% compared to non-T2DM individuals (OR = 1.52, 95% CI 1.25–1.85) (20). Other research also confirms this finding, with a meta-analysis of six observational studies involving all 2,453 patients. Compared to patients without T2DM or normal glucose levels, individuals with T2DM were more than twice as likely to develop surgical PUs (OR = 2.15, 95% CI: 1.62–2.84) (21). One potential explanation is that T2DM induces peripheral neuropathy, impairs sensory perception, and leads to prolonged insensitivity to compression, tissue necrosis, and nonhealing of affected areas. Peripheral neuropathy, in turn, has also been associated with neuropathic ulcers and diabetic foot ulcers (32–34). Another mechanism is that T2DM and high FG levels have been shown to reduce blood flow to the skin and underlying tissues. Poor circulation can result in decreased oxygen and nutrient delivery to these tissues, making them more vulnerable to damage from pressure and friction. Impaired blood flow also slows down the healing process of PUs once they have formed (35). Additionally, T2DM can cause chronic inflammation and reduced angiogenesis, further hindering the healing processes of PUs. In mice models of T2DM, reducing inflammation by increasing levels of several pro-healing growth factors has been shown to improve wound healing (36–38). Lastly, T2DM can reduce the formation of collagen, a protein essential for skin strength and elasticity. This can affect the skin’s ability to withstand pressure and shear forces, making it more susceptible to injury and increasing the likelihood of developing PUs (39). Moreover, T2DM and a longer duration of hyperglycemia can lead to metabolic imbalances, including altered protein and nutrient metabolism (40). These potential mechanisms can affect the body’s ability to generate new tissue and repair damaged skin. Adequate nutrition is critical for the wound healing of PUs, and individuals with poorly controlled T2DM and FI levels may be at greater risk of malnutrition (41, 42). Our findings also suggest a reciprocal causation between FG and PUs, elevated FG is often associated with insulin resistance, a condition where the body’s cells become less responsive to the effects of insulin and tissue damage and inflammation caused by PUs may trigger a stress response in the body, potentially affecting insulin sensitivity and metabolism (43). Therefore, high levels of FI and FG are closely associated with the metabolic response and disease progression in PUs.

It is worth noting that our study revealed an inverse association between salad/raw vegetable intake and PUs. Consistent with previous studies, the beneficial impact could be attribeted to components found in salad/raw vegetables, which are typically rich in vitamins, minerals, sufficient water, and antioxidants. These components play crucial roles in skin health, digestive health, regular bowel movements, and tissue repair (43–46). Recent evidence emphasizes that plant polyphenolic compounds, which are prevalent in the human diet and present in substances such as curcumin (47), apigenin (48), *Ocimum basilicum*, and *Trifolium pratense* extracts (49), confer various health benefits. These compounds are well-recognized for their potent antioxidant, antimicrobial, and anti-inflammatory properties. They neutralize free radicals by inhibiting key signaling pathways, including NF-κB, transforming growth factor-beta (TGF-*β*), and mitogen-activated protein kinase, while also enhancing the activity of antioxidant enzymes including superoxide dismutase (SOD), peroxidases, and catalase (50). Collectively, these effects support wound healing and skin barrier repair. Additionally, these polyphenolic compounds are crucial in recruiting specific cells to sites of inflammation and accelerating the overall healing process. Adequate nutrient supply can promote healthy skin and reduce the risk of PUs development. Diets with higher vegetable intake are generally more favorable for weight management. Maintaining an appropriate weight can alleviate the pressure on the skin, reducing the likelihood of skin damage. Certain components in vegetables have anti-inflammatory and immune-supporting properties, potentially helping to lower skin inflammation levels and mitigate tissue damage caused by inflammation (51).

The relationship between alcohol intake and the risk of developing PUs is a matter of debate, with varying results from different studies. Several studies have linked alcohol consumption to an increased risk of developing PUs (52, 53). However, other research has found that the consumption of alcohol was not significantly linked to the incidence of PUs (54, 55). Despite applying MR analysis to investigate the potential association between alcohol consumption and PUs, no causal association was shown in the study. Vegetables, abundant in Vitamin C and Quercetin, are commonly recommended as dietary components for the prevention or treatment of PUs (44, 56, 57).

Our study has several strengths and limitations. Firstly, this is the first study to investigate the causal relationship between T2DM, glycemic traits, dietary habits and PUs through a Two-sample MR analysis, which is not affected by confounders and reverse causation compared with observational studies. Additionally, we conducted multivariable MR analyses to disentangle the direct causal impacts of T2DM on PUs. Finally, multiple sensitivity analyses and IV strength evaluations were conducted to ensure the robustness and reliability of the results. However, there are also some limitations. Firstly, MR analysis is based on specific assumptions that cannot be verified. Secondly, the present study primarily involved individuals of European ancestry, which may limit the generalizability of our findings to other populations. Thirdly, while we explored the association between T2DM, salad/raw vegetable intake, FI, FG and PUs from a genetic perspective, the underlying mechanisms remain unclear and warrant further investigation.

## 5 Conclusion

This study found that salad/raw vegetabke intake associated with a reduced risk of PUs, while T2DM and FG were associated with an increased risk of PUs. Furthermore, FI and FG may play pivotal roles as downstream factors in PUs. This study also found that various dietary habits including alcoholic drinks per week, alcohol intake frequency, bread intake, cheese intake, water intake, cereal intake, non-oily fish intake, oily fish intake, beef intake, fresh fruit intake, coffee intake, pork intake, tea intake, processed meat intake, poultry intake, and cooked vegetable intake were not associated with PUs, which were robust to different analyses and rigorous pleiotropy testing.

## Data Availability

The original contributions presented in the study are included in the article/Supplementary material, further inquiries can be directed to the corresponding author.

## Footnotes

^1^ https://gwas.mrcieu.ac.uk/

^2^ http://magicinvestigators.org/downloads/

^3^ https://r9.finngen.fi/

^4^ http://www.nealelab.is/uk-biobank/

^5^ http://www.phenoscanner.medschl.cam.ac.uk

## Supplementary material

SUPPLEMENTARY FIGURE 1 Funnel plot in the Mendelian randomization analysis of T2DM, salad/raw vegetable intake, glycemic traits and Pressure ulcers. Funnel plot of T2DM **(A)**, Srvi **(B)**, and FG **(C)** on PUs and PUs on FG **(D)** and FI **(E)**. T2DM, Type 2 Diabetes Mellitus; Srvi, salad/raw vegetable intake; PUs, Pressure ulcers; FG, fasting Glucose; FI, Fasting Insulin.

SUPPLEMENTARY FIGURE 2 Forest plot in the Mendelian randomization analysis of T2DM, salad/raw vegetable intake, glycemic traits and Pressure ulcers. Forest plot of T2DM **(A)**, Srvi **(B)**, and FG **(C)** on PUs and PUs on FG **(D)** and FI **(E)**. T2DM, Type 2 Diabetes Mellitus; Srvi, salad/raw vegetable intake; PUs, Pressure ulcers; FG, fasting Glucose; FI, Fasting Insulin.
