# Supplementary figures and images for: Causal associations between type 2 diabetes mellitus, glycemic traits, dietary habits and the risk of pressure ulcers: univariable, bidirectional and multivariable Mendelian randomization
Source: Front Nutr. 2024 Oct 2;11:1375179. doi: 10.3389/fnut.2024.1375179 (PMC11480076; doi:10.3389/fnut.2024.1375179)

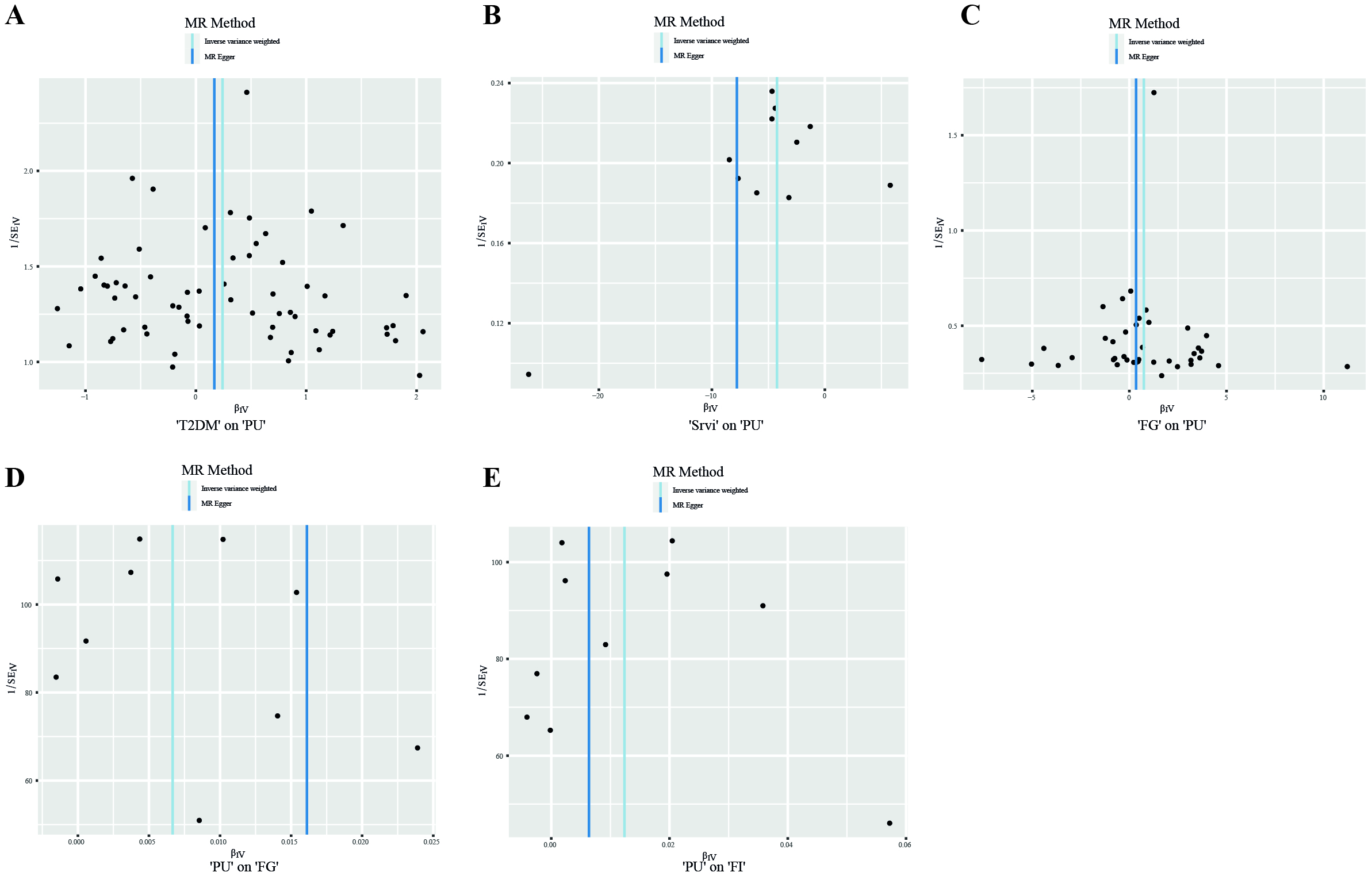

Supplement: SUPPLEMENTARY FIGURE 1 — Funnel plot in the Mendelian randomization analysis of T2DM, salad/raw vegetable intake, glycemic traits and Pressure ulcers. Funnel plot of T2DM (A), Srvi (B), and FG (C) on PUs and PUs on FG (D) and FI (E). T2DM, Type 2 Diabetes Mellitus; Srvi, salad/raw vegetable intake; PUs, Pressure ulcers; FG, fasting Glucose; FI, Fasting Insulin. [file Image_1.JPEG]

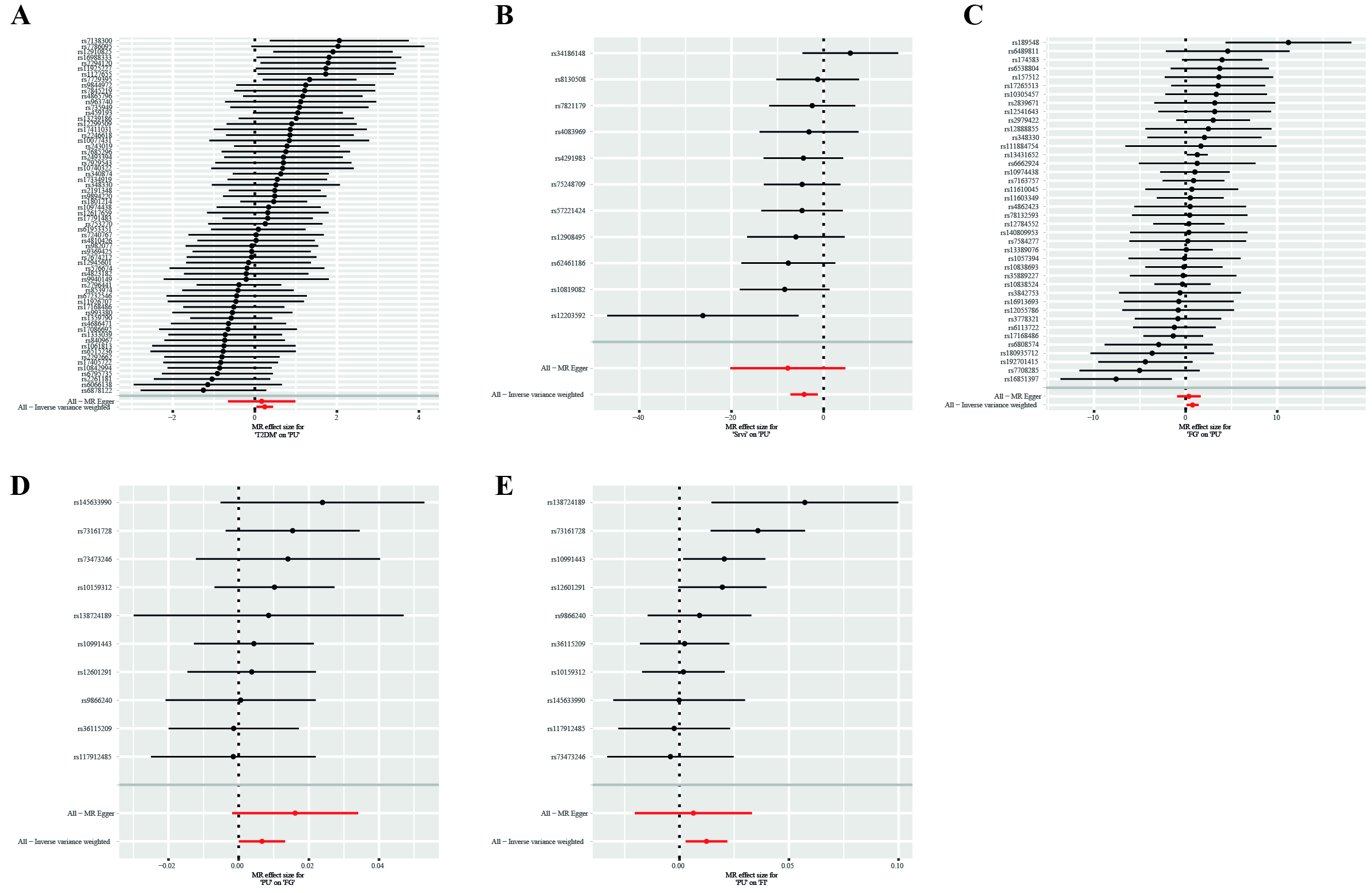

Supplement: SUPPLEMENTARY FIGURE 2 — Forest plot in the Mendelian randomization analysis of T2DM, salad/raw vegetable intake, glycemic traits and Pressure ulcers. Forest plot of T2DM (A), Srvi (B), and FG (C) on PUs and PUs on FG (D) and FI (E). T2DM, Type 2 Diabetes Mellitus; Srvi, salad/raw vegetable intake; PUs, Pressure ulcers; FG, fasting Glucose; FI, Fasting Insulin. [file Image_2.JPEG]
